# Efficacy analysis following polymer coated drug eluting stent and bare metal stent deployment for femoropopliteal arterial disease

**DOI:** 10.1177/17085381221126217

**Published:** 2022-09-07

**Authors:** Yousef Shehada, Theodosios Bisdas, Angeliki Argyriou, Giovanni Torsello, Nikolaos Tsilimparis, Efthymios Beropoulis, Konstantinos Stavroulakis

**Affiliations:** 1 Department of Vascular and Endovascular Surgery, 39612 St. Franziskus Hospital Muenster, Germany; 2 Department of Vascular Surgery, 69046 Athens Medical Center, Athens Greece; 3 Department of Vascular and Endovascular Surgery, Augusta Hospital Duesseldorf, Germany; 4 Department of Vascular and Endovascular Surgery, Ludwig-Maximilians-University Hospital Munich, Germany

**Keywords:** Mortality, paclitaxel, femoropopliteal, PAD, scaffolds

## Abstract

**Objectives:**

The objective is to assess the performance of the Eluvia polymer coated drug eluting stent (DES) compared to a bare metal stent (BMS) platform in patients with femoropopliteal arterial disease.

**Methods:**

This is a retrospective, single-center analysis. Patients treated with the Eluvia DES (group Eluvia) or the EverFlex BMS (group BMS) for femoropopliteal disease between January 2013 and December 2019 were included. Primary measure outcome of this analysis was the overall mortality. The PTX specific mortality, the primary patency, the amputation free survival (AFS), and the target lesion revascularization (TLR) rates were additionally evaluated.

**Results:**

A total of 124 patients were treated by BMS deployment, while the Eluvia platform was preferred in 75 subjects. In both groups the majority presented with lifestyle limiting claudication (BMS: 84% vs Eluvia: 73%, *p* = 0.73). Chronic total occlusions were more frequent in patients treated by BMS (BMS: 71% vs Eluvia: 84%, *p* = 0.027), whereas the calcification burden (BMS: 81% vs Eluvia: 76%, *p* = 0.43) and the median lesion length (in mm, IQR) (BMS: 160 (100 to 240) vs Eluvia: 140 (80 to 229), *p* = 0.17) were comparable. At 24 months, the overall survival (BMS: 93% vs Eluvia: 89%, hazard ratio (HR): 1.20, 95% confidence interval (CI): 0.55 to 2.64, *p* = 0.64) and the PTX specific survival (BMS: 95% vs Eluvia: 95%, HR: 1.28, 95% CI: 0.41 to 4.02, *p* = 0.67) did not differ significantly between the two platforms. No significant difference was observed regarding the 24 months primary patency rate (BMS: 66% vs Eluvia: 78%, HR: 0.65, 95% CI: 0.37 to 1.15, *p* = 0.18), the freedom from TLR (BMS: 83% vs Eluvia: 89%, HR: 0.81, 95% CI: 0.39 to 1.68, *p* = 0.572), and the AFS (BMS: 93 vs Eluvia: 89%, HR: 1.20, 95% CI: 0.55 to 2.64). The Cox regression analysis revealed a higher mortality risk among patients with chronic limb-threatening ischemia (CLTI) (HR: 3.14, 95% CI: 1.61 to 6.14, *p* = 0.008), chronic obstructive pulmonary disease (COPD) (HR: 4.65, 95% CI: 2.14 to 10.09, *p* = 0.001), in octagenerians (HR: 4.40, 95% CI: 1.92 to 10.44, *p* = 0.005), and in patients not on statins at baseline (HR: 2.44, 95% CI: 1.19 to 4.99, *p*=0.014).

**Conclusions:**

In this cohort, the use of the Eluvia DES did not increase the risk for mortality compared to BMS deployment. CLTI, COPD, advanced age, and the lack of statin therapy at baseline were associated with a higher risk for death.

## Introduction

Endovascular therapy has evolved rapidly over the last years and is currently considered the initial approach for the majority of patients with peripheral arterial disease (PAD).^1^ Historically, plain angioplasty was the treatment of choice for infrainguinal atherosclerosis.^2^ However, the risk for neointimal hyperplasia and consequently for restenosis, clinical failure, and repeated interventions was rather high.^3,4^ Paclitaxel (PTX), a lipophilic antineoplastic agent, can be locally delivered in the arterial wall either with drug coated balloons (DCB) or drug eluting stents (DES) in order to inhibit neointimal hyperplasia. Numerous randomized control trials (RCTs) showed higher patency rates after the application of PTX coated devices compared to their non-coated competitors.^5^ Additionally, the use of DCBs and DES was associated with promising results in challenging real-world cohorts with a long-term sustained clinical benefit.^1,6^

In December 2018, a study-level meta-analysis of 28 RCTs with 4663 patients questioned the safety of PTX for peripheral interventions. Katsanos et al.^7^ reported an increased risk for all-cause mortality, which manifests ≥2 years after the use of DCB or DES. Following the initial publication, the Food and Drug Administration (FDA) performed its own meta-analysis showing that PTX-related devices were associated with increased mortality 3 years after the index procedure.^8^ Moreover, an individual patient data meta-analysis was published from the Vascular InterVentional Advances (VIVA) physicians and found an absolute 4.6% risk for mortality after PTX exposure.^9^ On the other hand, data from pragmatic real-world cohorts could not confirm this finding and more recently the interim analysis of the randomized registry-based SWEDEPAD trial did not reveal any difference between PTX coated and uncoated devices.^10-13^An important parameter of the FDA analysis is that a mortality class effect could not be determined.^8^ Thus, the safety profile of the different PTX coated devices has to be evaluated separately.

The Eluvia (Boston Sci. MA, USA) is a DES platform which uses a dual-layer coating, which enables the sustained release of PTX over the first 12 months after the stent deployment.^14^ Initial reports showed promising mid-term results for the treatment of complex femoropopliteal lesions.^1,14^ However, the Eluvia platform was included neither in the meta-analysis published from Katsanos et al. nor in the analysis of the FDA, while the device received an FDA-approval based on a noninferiority trial compared with the Zilver PTX DES (Cook Medical, Bloomington, IN).^15,16^ In this context, there is a relevant gap of data regarding the safety profile of this device compared to non-coated scaffolds. The aim of this study is to assess the safety and the efficacy of the Eluvia DES compared to a bare metal stent (BMS) platform in patients with symptomatic femoropopliteal disease.

## Methods

This is a single-center, retrospective analysis, performed in line with the requirements of the local ethics committee and adhering to the Declaration of Helsinki. All patients with symptomatic PAD who underwent endovascular treatment of a femoropopliteal lesion between January 2013 and December 2019, with the Eluvia DES or with the EverFlex (Medtronic, Plymouth, Minn) BMS were included into this study. The main criterion for the stent implantation was recoil or flow-limiting dissection after plain angioplasty. The selection of the deployed scaffold (DES or BMS) was based on the preference of the treating physician. This study followed the reporting guidelines from the STROBE (Strengthening the Reporting of Observational Studies in Epidemiology) statement for cohort studies.^17^

In order to solely evaluate the impact of the Eluvia platform on the mortality risk we excluded all cases with bailout stenting after DCB angioplasty. Moreover, all patients with previous or concomitant PTX DCB/DES treatment for additional lesions or the same lesion as well as patients with PTX treatment for coronary disease were excluded from our analysis. Patients previously or simultaneously treated with non-PTX drug coated devices for below the knee disease (Sirolimus, Everolimus etc.) were not excluded.

All patients underwent a thorough clinical examination at baseline. Patient demographics, imaging, and clinical data were prospectively collected and retrospectively analyzed. Follow-up examinations were scheduled at 6 and 12 and 24 months after the index procedure. The patency of the treated vessels was assessed by duplex ultrasound at each follow-up. In cases of clinical worsening, an angiography was performed.

Dual antiplatelet therapy with aspirin (ASA) (100 mg/d) and clopidogrel (75 mg/d) was routinely prescribed for 3 months, followed by ASA or clopidogrel monotherapy lifelong. Patients previously taking warfarin or oral anticoagulants were maintained on the anticoagulant with an additional antiplatelet therapy for 3 months after the procedure. A statin therapy was suggested in all patients.

### Endpoints and definitions

The primary measure outcome of this study was the overall mortality. A PTX specific survival analysis was also performed. For the specific survival evaluation, the analysis was censored in the event of PTX treatment at follow-up regardless indication. Secondary outcomes were primary patency rate, freedom from clinically driven target lesion revascularization (CD-TLR), and freedom from major amputation. Primary patency was defined as freedom from significant restenosis or occlusion without any re-intervention.

Significant restenosis was indicated by a >2.0 peak systolic velocity ratio calculated as the peak systolic flow velocity in the lesion divided by the peak systolic velocity 1 cm proximal to the lesion. Amputation-free survival was defined as the time until a major amputation of the index limb and/or death of any cause, whichever occurred first. A major amputation was defined as any above-ankle amputation. The degree of calcification was graded based on the arterial wall calcium deposits observed during fluoroscopy based on the suggested Peripheral Arterial Calcium Scoring Scale (PACSS). Grade 0 represents the lack of visible calcium at the target lesion, grade 1 refers to unilateral calcification shorter than 5 cm, grade 2 refers to unilateral wall calcification longer than 5 cm, grade 3 shows the presence of bilateral wall calcification shorter than 5 cm, and finally grade 4 is defined as bilateral wall calcification with calcium extension longer than 5 cm.^18^ The cutoff of 15 cm was used to define long lesions based on the Consensus Definitions From Peripheral Academic Research Consortium (PARC)^19^

### Statistical analysis

For the statistical analysis and graphics, the MedCalc Statistical Software (version 12.4.0.0; MedCalc Software, Ostend, Belgium) was used. Continuous variables are presented as means ± standard deviation or median (interquartile range), while categorical data are given as the counts. Continuous numeric variables were compared by Student *t* test for paired samples or Wilcoxon test according to their distribution (D-Agostino-Pearson test). Cumulative survival, PTX specific survival, primary patency, as well as freedom from TLR and amputation-free-survival were estimated using the Kaplan-Meier method. A univariate analysis was performed for patients with patent stents versus patients with patency loss to identify statistically significant differences between the groups. Similar analysis was performed in the total cohort regarding mortality; these variables were included in Cox regression analyses to determine risk factors for patency loss and death, respectively. The threshold of statistical significance was *p* ≤ 0.05.

## Results

During the study period, 199 patients met the inclusion criteria. In 124 patients, the EverFlex BMS was preferred, while the Eluvia platform was used in 75 subjects. In both groups, most patients were male (59 vs 59%, *p* = 0.97) and presented with lifestyle limiting claudication, with a non-significant trend to higher rate of chronic limb-threatening ischemia (CLTI) in the Eluvia group (16 vs 27%, *p* = 0.073). Table 1 summarizes the baseline characteristics of the cohort. A chronic total occlusion (CTO) was more frequently observed in patients treated by BMS (84 vs 71%, *p* = 0.02), while the median lesion length (in mm, IQR) of the two groups was comparable (BMS: 160 (100–240) vs Eluvia: 140 (80–229), *p*= 0.17). Although the presence of calcified disease did not differ significantly (BMS: 81% vs Eluvia: 76%, *p* = 0.43), a PACSS grade 1 (BMS: 44% vs Eluvia: 25%, *p* = 0.11) and 2 (BMS: 15% vs Eluvia: 2%, *p* = 0.007) was more common in the BMS subgroup and grade 3 (BMS: 12% vs Eluvia: 35%, *p* = 0.002) in the Eluvia group. Table 2 provides an overview of the lesion characteristics of the treated patients.

**Table 1.** Demographics.

Characteristic	Group BMS	Group Eluvia	*p*-value
Total	124	75	—
Males	73 (59%)	44 (59%)	0.977
Age (in years, median, IQR)	74 (65 to 80)	72 (66 to 80)	0.628
Rutherford classes			
Class 2	21 (17%)	0	0.002
Class 3	79 (64%)	54 (72%)	
Class 4	8 (7%)	4 (5%)	
Class 5	15 (12%)	14 (19%)	
Class 6	1 (0.8%)	3 (4%)	
Chronic limb-threatening ischemia	24 (16%)	21 (27%)	0.001
Arterial hypertension	107 (86%)	83 (84%)	0.658
Dyslipidemia	114 (92%)	63 (84%)	0.084
Diabetes mellitus	37 (30%)	26 (35%)	0.479
Congestive heart disease	49 (40%)	29 (39%)	0.905
Obesity	30 (24%)	25 (33%)	0.163
COPD	19 (15%)	11 (15%)	0.901
Cerebrovascular disease	31 (25%)	23 (31%)	0.385
End-stage renal disease	0	2 (3%)	0.068
Chronic kidney disease	26 (21%)	18 (24%)	0.618
Current tobacco use	47 (38%)	20 (27%)	0.105
Previous peripheral bypass	1 (1%)	0	0.437
Previous vascular intervention	17 (14%)	4 (5%)	0.061

**Table 2.** Anatomic and procedural characteristics.

Characteristic	Group BMS	Group Eluvia	*p*-value
Chronic total occlusion	104 (84%)	53 (71%)	**0.027**
Calcification	100 (81%)	57 (76%)	0.438
PACSS 1	44 (44%)	14 (25%)	0.011
PACSS 2	15 (15%)	1 (2%)	0.007
PACSS 3	12 (12%)	20 (35%)	0.002
PACSS 4	29 (29%)	21 (38%)	0.468
Median lesion length (in mm, IQR)	160 (100 to 240)	140 (80 to 229)	0.172
Lesion length ≥150 mm	65 (52%)	35 (47%)	0.433
Lesion length ≥250 mm	23 (19%)	17 (23%)	0.484
Run-off vessels ≥2	101 (82%)	58 (77%)	0.484
Median stent length (in mm, IQR)	215 (150 to 300)	135 (80 to 230)	<0.0001
Number of stents			
1	57 (46%)	42 (56%)	0.232
2	54 (44%)	27 (36%)	
3	12 (10%)	5 (7%)	
4	1 (1%)	1 (1%)	
Anatomic position of stenting			
Proximal SFA	70 (57%)	26 (35%)	0.003
Mid SFA	96 (77%)	44 (59%)	0.005
Distal SFA	106 (86%)	61 (81%)	0.441
P1	40 (32%)	28 (37%)	0.466
P2	17 (14%)	6 (8%)	0.223
P3	6 (5%)	0	0.054
Full lesion cover	122 (98%)	63 (84%)	0.0001
Spot stenting	2 (2%)	9 (12%)	0.002
Additional procedures	35 (28%)	22 (29%)	0.867

**Figure 1.**
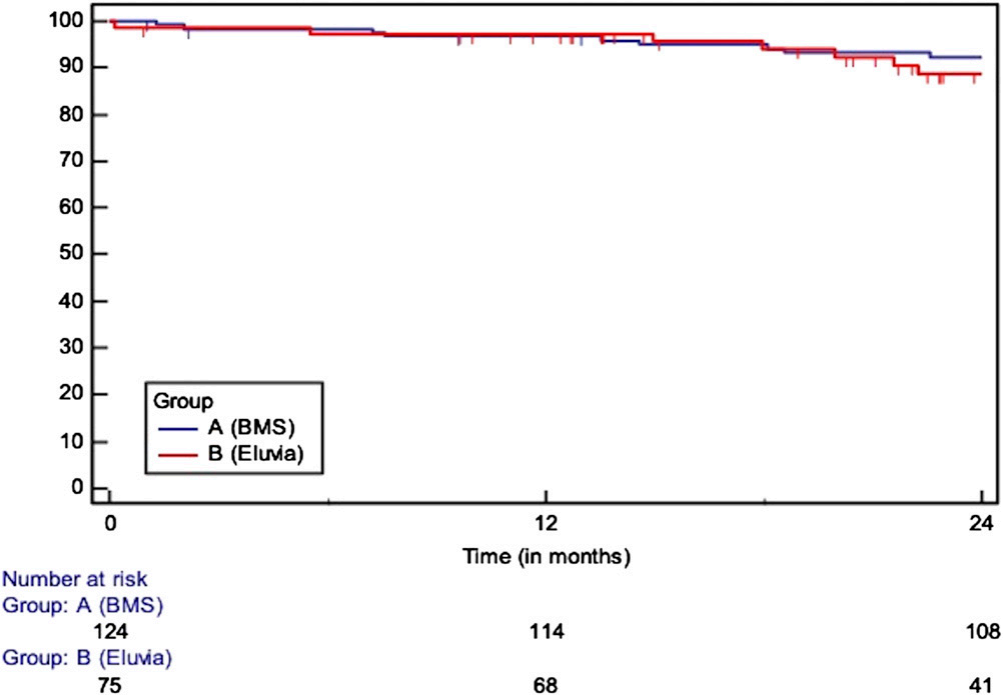
Survival (HR: 1.20, 95% CI: 0.55 to 2.64, *p* = 0.644), at 12 months: 97 vs 98%, at 24 m: 93% vs 89%)/SE>10% at 25.18 months.

**Figure 2.**
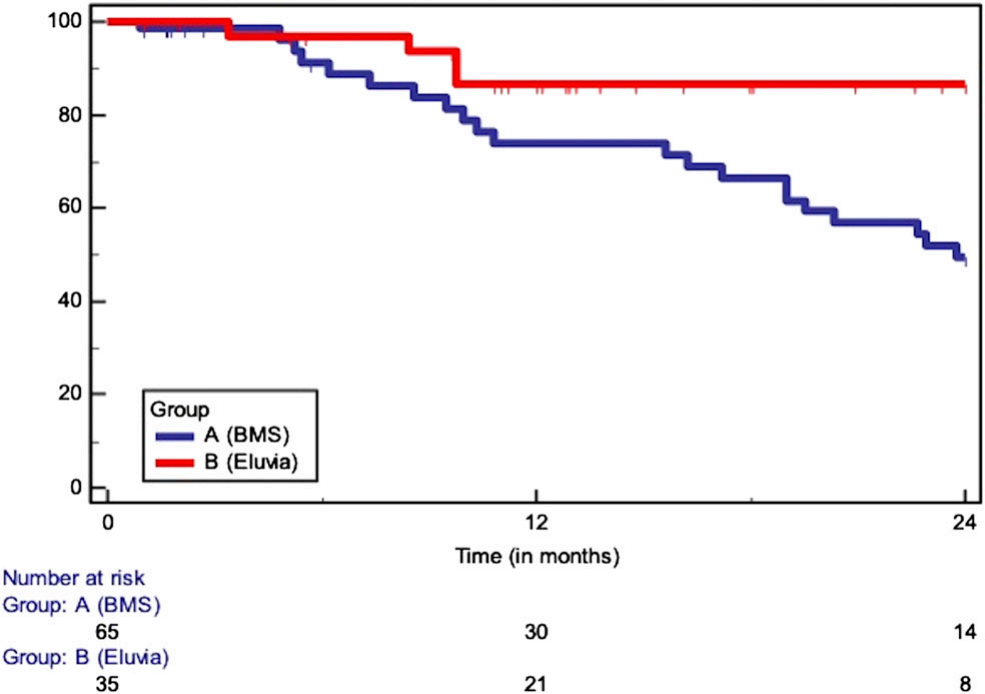
Primary patency in lesions longer than 150 mm (at 12 m: 73 vs 87%, at 24 m: 50 vs 87%, HR: 2.63, 95% CI: 1.29 to 5.36)/SE>10% at 24.79 months.

The median follow-up (in months, IQR) was longer in patients treated by the EverFlex BMS (BMS: 63, 46 to 77 vs Eluvia: 28, 19 to 42, *p* < 0.0001). At 24 months (Table 3), the overall survival (BMS: 93% vs Eluvia: 89%, HR: 1.20, 95% CI: 0.55 to 2.64, *p* = 0.64) (Figure 1) and the PTX specific survival (BMS: 95% vs Eluvia: 95%, HR: 1.28, 95% CI: 0.41 to 4.02, *p* = 0.67) did not differ significantly between the coated and the uncoated scaffold. In both groups, the freedom from major amputation at 24 months amounted to 100%. No statistically notable difference was shown regarding the AFS at 24 months following the deployment of DES or BMS (BMS: 93% vs Eluvia: 89%, HR: 1.20, 95% CI: 0.55 to 2.64). The freedom from CD-TLR was 88% (BMS) vs 96% (Eluvia) at 12 months and 83% (BMS) vs 89% (Eluvia) at 24 months (HR: 0.81, 95% CI: 0.39 to 1.68, *p* = 0.572). Similarly, the primary patency at 12 months (BMS: 80% vs Eluvia: 93%) and 24 months (BMS: 66% vs Eluvia: 78%) did not differ significantly between the two groups (HR: 0.65, 95% CI: 0.37 to 1.15, *p* = 0.184). However, the deployment of the Eluvia DES was associated with higher patency rate (BMS: 50% vs Eluvia: 87%, HR: 2.63, 95% CI: 1.29 to 5.36) but comparable to CT-TLR rates (BMS: 74% vs Eluvia: 88%, HR: 1.76, 95% CI: 0.73 to 4.22, *p* = 0.206) at 24 months in lesions longer than 150 mm (Figure 2).

**Table 3.** Overview of the 24-months outcomes between the two groups.

Outcome	Group BMS, %	Group Eluvia, %	HR, 95% CI	*p*- value
Overall survival	93	89	1.20, 0.55 to 2.64	0.644
PTX-specific survival	95	95	1.28, 0.41 to 4.02	0.672
Amputation-free survival	93	89	1.20, 0.55 to 2.64	0.644
Overall freedom from CD-TLR	83	89	0.81, 0.39 to 1.68	0.572
Overall freedom from restenosis	66	78	0.65, 0.37 to 1.15	0.184
Freedom from CD-TLR in lesions >150 mm	74	88	1.76, 0.73 to 4.22	0.206
Freedom from restenosis in lesions >150 mm	50	87	2.63, 1.29 to 5.36	0.036

The Cox regression analysis revealed a higher mortality risk among patients with chronic limb-threatening ischemia (CLTI) (HR: 3.14, 95% CI: 1.61 to 6.14, *p* = 0.008), chronic obstructive pulmonary disease (COPD) (HR: 4.65, 95% CI: 2.14 to 10.09, *p* = 0.001), in octagenerians (HR: 4.40, 95% CI: 1.92 to 10.44, *p* = 0.005), and in patients not on statin therapy at baseline (HR: 2.44, 95% CI: 1.19 to 4.99, *p* = 0.014) **(**Table 4)**.** In lesions longer than 150 mm, the presence of PACSS lesions 1 (HR: 0.34, 95% CI: 0.14 to 0.82, *p*=0.01) and the use of the Eluvia DES (HR: 0.3, 95% CI: 0.10 to 0.91, *p* = 0.03) reduced the risk for restenosis, while a non-significant trend for patency loss was observed in CTOs (HR: 6.73, 95% CI: 0.87 to 51.9, *p* = 0.06) (Table 5).

**Table 4.** Cox regression analysis of risk factors for mortality during surveillance in the overall cohort.

Variable	Hazard ratio	95% CI	*p*-value
Chronic limb-threatening ischemia	3.14	1.61 to 6.14	0.0008
Octogenarians	4.49	1.92 to 10.44	0.0005
COPD	4.65	2.14 to 10.09	0.0001
No statins preoperatively	2.44	1.19 to 4.99	0.0147

**Table 5.** Cox regression analysis of risk factors for restenosis in lesions longer than 150 mm.

Covariate	HR	95% CI of HR
Eluvia DES	0.3043	0.1012 to 0.9145
CTO^a^	6.7348	0.8739 to 51.9036
PACSS^b^ 1	0.3493	0.1474 to 0.8279
PACSS 2	0.7122	0.2404 to 2.1099
PACCS 3	0.3105	0.0389 to 2.4810
P_SFA^c^	1.0180	0.2237 to 4.6337
M_SFA^d^	0.3019	0.0272 to 3.3474

^a^CTO: chronic total occlusion.

^b^PACSS: Peripheral Arterial Calcification Scoring Scale.

^c^P_SFA: proximal superficial femoral artery.

^d^M_SFA: middle superficial femoral artery.

## Discussion

The increased mortality risk, observed after the use of PTX coated devices, had a significant impact on the treatment of patients with PAD and prompted a debate among vascular specialists.^16^ The Eluvia DES was not included in the initial meta-analysis and till now there is no available data regarding the safety profile of this polymer coated DES compared to non-PTX coated devices.^1,7^ In this cohort, the use of the Eluvia DES was not associated with a higher risk for death, while its clinical performance was comparable to the EverFlex BMS. A benefit in terms of increased patency was shown in lesions longer than 150 mm. CLTI, COPD, advanced age, and the lack of statin therapy at baseline were identified as independent risk factors for mortality.

In December 2018, a meta-analysis of 28 RCTs described an increased mortality rate after the use of PTX coated DCBs and DESs for femoropopliteal PAD.^7^ Many concerns were raised regarding the design of this study, the heterogeneity of the mortality association across the trials and the lack of patient level data. However, two additional studies from the FDA and the VIVA physicians also reported a higher risk for death after the application of PTX.^8,9^ On the contrary, recent meta-analyses, studies of large-scale registries and single-center experiences could not confirm this finding.^10–13,20–23^

Although RCTs are considered the most scientifically rigorous clinical study design, patients included in prospective trials are highly selected, carry a different atherosclerotic profile and have a decreased comorbidity compared to real-world pragmatic cohorts.^22,24^ Unlike RCTs, registries and insurance claim data provide information on a more generalizable group of patients and physician practices.^22^ Nonetheless, the published registries were not without limitations. No device specific recommendations could be made, the lesions characteristics could not be analyzed, and the use of PTX for coronary or peripheral interventions prior to the index procedure was unknown, while in case of TLR or repeated intervention, the secondary application of PTX was not assessed. In our study, we excluded patients previously or concomitantly treated by PTX and we additionally performed a PTX specific analysis for repeated interventions. Both the overall mortality and the PTX specific mortality did not differ between the two groups.

The discrepancy between the initially published meta-analysis, the VIVA and FDA evaluation and the registry data, mirrors the differences between the treated cohorts. In this study, although the Eluvia did not influence the risk for death, the presence of CLTI, COPD, and advance age were independent risk factors for mortality. Moreover, like previous observations, the lack of statin therapy was identified as risk factor highlighting the need for aggressive secondary prophylaxis.^25^ Of note, these groups of patients are usually excluded from prospective trials, while the studies included in the meta-analysis of Katsanos et al.^7^ did not have a standardized secondary prophylaxis protocol and could not provide any information regarding the medications of the included patients.

Regarding the indication for DES deployment in femoropopliteal lesions, a benefit was shown in this cohort only for lesions longer than 150 mm. In the Zilver PTX randomized trial, higher patency rates for relatively short lesions (average lesion length was ≈65±40 mm) were observed following the deployment of the Zilver PTX DES (Cook Medical, Bloomington, IN); however, in the Bare Metal Stent versus Paclitaxel Eluting Stent in the Setting of Primary Stenting of Intermediate-Length Femoropopliteal Lesions (BATTLE) trial, the Zilver PTX stent failed to show superiority over a BMS platform stent in freedom from restenosis at 1 year (mean lesion length was 7.6 ± 4.1 cm).^3,26^ In a mixed cohort of polymer and non-polymer coated DES, Saratzis et al.^27^ reported comparable results between DES and interwoven stents for femoropopliteal disease. The ongoing EMINENT trial (NCT02921230) will assess the performance of the Eluvia DES over various commercially available BMS. Patients will be followed for 5 years. Although the study will provide valuable information regarding the efficacy of DES to inhibit restenosis, the exclusion of individuals with advanced ischemia (Rutherford class 5 and 6) might limit its applicability in a real-world scenario.

Given the promising performance of the Eluvia DES in complex disease, the high risk for restenosis with BMS in long lesions, and the results of this cohort, the preferable use of the Eluvia in longer lesions when a scaffold is needed seems reasonable.^1,28^ This approach has been adopted from many physicians as data from the Vascular Quality Initiative registry from 2010 to 2017 showed that DES were preferred over BMS for the treatment of longer lesions.^29^

## Limitations

This study carries the well-known limitations of retrospective registries. Despite the comparable baseline characteristics of the two groups, the lack of randomization remains an important limitation of this analysis. Finally, a higher patient volume and a longer follow-up (up to 5 years) might be needed to reveal a late mortality signal. Regarding the efficacy of both devices to inhibit restenosis, the median stent length in the BMS group was 215 mm vs 135 mm in the PTX eluting stent group. Also, chronic total occlusion frequency was significantly higher in the BMS group. Similarly, the BMS group had significantly higher rates of proximal and mid SFA stenting versus PTX stent. This might lead to a possible bias against the BMS group. Additionally, the selection of the used platforms was at the discretion of the treating physicians. The Eluvia DES was introduced in 2016 and patients treated prior to 2016 were mainly treated with BMS deployment. Nonetheless, after the introduction of the Eluvia platform, this scaffold was preferred. Finally, we did not perform any PTX dose dependent analysis, although the current body of literature does not suggest any dose dependent mortality effect.

## Conclusions

Among patients undergoing femoropopliteal peripheral endovascular intervention with the Eluvia DES, there was no increased risk of 24 months, all-cause mortality. CLTI, COPD, advance age, and the lack of statin therapy were identified as independent risk factors for death. Comparable clinical outcomes, patency, and CD-TLR rates were observed following the use of the Eluvia and the EverFlex platforms in the overall cohort, while the use of DES reduced the restenosis rate in lesions longer than 150 mm.

## References

[1] Stavroulakis K Torsello G Bosiers M , et al. 2-Year outcomes of the eluvia drug-eluting stent for the treatment of complex femoropopliteal lesions. JACC Cardiovasc Interv 2021; 14(6): 692–701. 33736776 10.1016/j.jcin.2021.01.026

[2] Adam DJ Beard JD Cleveland T , et al. BASIL trial participants. Bypass versus angioplasty in severe ischemia of the leg (BASIL): multicentre, randomised controlled trial. Lancet 2005; 366(9501): 1925–1934. 16325694 10.1016/S0140-6736(05)67704-5

[3] Dake MD Ansel GM Jaff MR , et al. Durable clinical effectiveness with paclitaxel-eluting stents in the femoropopliteal artery: 5-Year results of the Zilver PTX Randomized Trial. Circulation 2016; 133(15): 1472–1483. 26969758 10.1161/CIRCULATIONAHA.115.016900 PMC4823823

[4] Laird JR Katzen BT Scheinert D , et al. Nitinol stent implantation versus balloon angioplasty for lesions in the superficial femoral ar- tery and proximal popliteal artery: twelve-month results from the RESILIENT randomized trial. Circ Cardiovasc Interv 2010; 3: 267–276. 20484101 10.1161/CIRCINTERVENTIONS.109.903468

[5] Katsanos K Spiliopoulos S Paraskevopoulos I , et al. Systematic Review and Meta-analysis of Randomized Controlled Trials of Paclitaxel-Coated Balloon Angioplasty in the Femoropopliteal Arteries: Role of Paclitaxel Dose and Bioavailability. J Endovasc Ther 2016; 23(2): 356–370. 26823485 10.1177/1526602815626557

[6] Torsello G Stavroulakis K Brodmann M , et al. PACT global investigators. three-year sustained clinical efficacy of drug-coated balloon angioplasty in a real-world femoropopliteal cohort. J Endovasc Ther 2020; 27(5): 693–705. 32583749 10.1177/1526602820931477 PMC7545651

[7] Katsanos K Spiliopoulos S Kitrou P , et al. Risk of death following application of paclitaxel-coated balloons and stents in the femoropopliteal artery of the leg: a systematic review and meta-analysis of randomized controlled trials. J Am Heart Assoc 2018; 7(24): e011245. 30561254 10.1161/JAHA.118.011245 PMC6405619

[8] Dan K Shlofmitz E Khalid N , et al. Paclitaxel-related balloons and stents for the treatment of peripheral artery disease: Insights from the Food and Drug Administration 2019 Circulatory System Devices Panel Meeting on late mortality. Am Heart J 2020; 222: 112–120. 32028137 10.1016/j.ahj.2019.12.012

[9] Rocha-Singh KJ Duval S Jaff MR , et al. VIVA Physicians, Inc. Mortality and Paclitaxel-Coated Devices: An Individual Patient Data Meta-Analysis. Circulation 2020; 141(23): 1859–1869. 32370548 10.1161/CIRCULATIONAHA.119.044697 PMC8029645

[10] Secemsky EA Kundi H Weinberg I , et al. Association of survival with femoropopliteal artery revascularization with drug-coated devices. JAMA Cardiol 2019; 4(4): 332–340. 30747949 10.1001/jamacardio.2019.0325 PMC6484791

[11] Freisinger E Koeppe J Gerss J , et al. Mortality after use of paclitaxel-based devices in peripheral arteries: a real-world safety analysis. Eur Heart J 2020; 41(38): 3732–3739. 31593987 10.1093/eurheartj/ehz698 PMC7666867

[12] Donas KP Sohr A Pitoulias GA , et al. Long-term mortality of matched patients with intermittent claudication treated by high-dose paclitaxel-coated balloon versus plain balloon angioplasty: a real-world study. Cardiovasc Intervent Radiol 2020; 43(1): 2–7. 31502025 10.1007/s00270-019-02329-z

[13] Nordanstig J James S Andersson M , et al. Mortality with paclitaxel-coated devices in peripheral artery disease. N Engl J Med 2020; 383(26): 2538–2546. 33296560 10.1056/NEJMoa2005206

[14] Bisdas T Beropoulis E Argyriou A , et al. 1-Year all-comers analysis of the eluvia drug-eluting stent for long femoropopliteal lesions after suboptimal angioplasty. JACC Cardiovasc Interv 2018; 11(10): 957–966. 29798772 10.1016/j.jcin.2018.03.046

[15] Gray WA Keirse K Soga Y , et al. IMPERIAL investigators. A polymer-coated, paclitaxel-eluting stent (Eluvia) versus a polymer-free, paclitaxel-coated stent (Zilver PTX) for endovascular femoropopliteal intervention (IMPERIAL): a randomised, non-inferiority trial. Lancet 2018; 392(10157): 1541–1551. 30262332 10.1016/S0140-6736(18)32262-1

[16] Schneider PA Varcoe RL Secemsky E , et al. Update on paclitaxel for femoral-popliteal occlusive disease in the 15 months following a summary level meta-analysis demonstrated increased risk of late mortality and dose response to paclitaxel. J Vasc Surg 2021; 73(1): 311–322. 32890719 10.1016/j.jvs.2020.07.093 PMC8076887

[17] von Elm E Altman DG Egger M , et al. The Strengthening the Reporting of Observational Studies in Epidemiology (STROBE) Statement: guidelines for reporting observational studies. Int J Surg 2014; 12: 1495–1499. 25046131 10.1016/j.ijsu.2014.07.013

[18] Patel MR Conte MS Cutlip DE , et al. Evaluation and treatment of patients with lower extremity peripheral artery disease: consensus definitions from Peripheral Academic Research Consortium (PARC). J Am Coll Cardiol 2015; 65(9): 931–941. 25744011 10.1016/j.jacc.2014.12.036 PMC4874808

[19] Rocha-Singh KJ Zeller T Jaff MR . Peripheral arterial calcification: prevalence, mechanism, detection, and clinical implications. Catheter Cardiovasc Interv 2014; 83: e212. 24402839 10.1002/ccd.25387 PMC4262070

[20] Gutierrez JA Rao SV Jones WS , et al. Survival and causes of death among veterans with lower extremity revascularization with paclitaxel-coated devices: insights from the veterans health administration. J Am Heart Assoc 2021; 10(4): e018149. 33554613 10.1161/JAHA.120.018149 PMC7955346

[21] Kim TI Kiwan G Mohamedali A , et al. Outcomes of treatment with paclitaxel-coated devices for peripheral arterial disease. J Vasc Surg 2021; 73(3): 911–917. 33038480 10.1016/j.jvs.2020.08.146

[22] Lottes AE Whatley EM Royce SM , et al. Important considerations for trials for peripheral arterial disease: Lessons learned from the paclitaxel mortality signal: A report on behalf of the registry assessment for peripheral interventional Devices (RAPID) Paclitaxel Pathways Program. Am Heart J 2021; 232: 71–83. 33157067 10.1016/j.ahj.2020.10.070

[23] Dinh K Limmer AM Chen AZL , et al. Mortality rates after paclitaxel-coated device use in patients with occlusive femoropopliteal disease: an updated systematic review and meta-analysis of randomized controlled trials. 2021: 15266028211023505. 10.1177/15266028211023505 34106028

[24] Ansel GM Brodmann M Keirse K , et al. PACT Global Study Investigators. Drug-Coated Balloon Treatment of Femoropopliteal Lesions Typically Excluded From Clinical Trials: 12-Month Findings From the IN.PACT Global Study. J Endovasc Ther 2018; 25(6): 673–682.; IN. 30280648 10.1177/1526602818803119 PMC6238185

[25] Stavroulakis K Borowski M Torsello G CRITISCH collaborators , et al. Association between statin therapy and amputation-free survival in patients with critical limb ischemia in the CRITISCH registry. J Vasc Surg 2017; 66(5): 1534–1542. 28807382 10.1016/j.jvs.2017.05.115

[26] Gouëffic Y Sauguet A Desgranges P , et al. A Polymer-Free Paclitaxel-Eluting Stent Versus a Bare-Metal Stent for De Novo Femoropopliteal Lesions: The BATTLE Trial. JACC Cardiovasc Interv 2020; 13(4): 447–457. 32081238 10.1016/j.jcin.2019.12.028

[27] Saratzis A Rudarakanchana N Patel S , et al. Interwoven Nitinol Stents versus Drug Eluting Stents in the Femoro-Popliteal Segment: A Propensity Matched Analysis. Eur J Vasc Endovasc Surg 2019; 58(5): 719–727. 31500990 10.1016/j.ejvs.2019.06.012

[28] Bosiers M Deloose K Callaert J , et al. Results of the Protégé EverFlex 200-mm-long nitinol stent (ev3) in TASC C and D femoropopliteal lesions. J Vasc Surg 2011; 54(4): 1042–1050. 21636239 10.1016/j.jvs.2011.03.272

[29] Mohapatra A Saadeddin Z Bertges DJ , et al. Nationwide trends in drug-coated balloon and drug-eluting stent utilization in the femoropopliteal arteries. J Vasc Surg 2020; 71(2): 560–566. 31405761 10.1016/j.jvs.2019.05.034 PMC7007839

